# Examining the assumptions of AI hiring assessments and their impact on job seekers’ autonomy over self-representation

**DOI:** 10.1007/s00146-023-01783-1

**Published:** 2023-10-21

**Authors:** Evgeni Aizenberg, Matthew J. Dennis, Jeroen van den Hoven

**Affiliations:** 1 AiTech Interdisciplinary Research Program on Meaningful Human Control Over AI, Delft University of Technology, Delft, The Netherlands; 2 Department of Intelligent Systems, Delft University of Technology, Delft, The Netherlands; 3 Present Address: Human Centered Design Group, Department of Design, Production and Management, University of Twente, Enschede, The Netherlands; 4 Philosophy and Ethics Group, Department of Industrial Engineering and Innovation Sciences, Eindhoven University of Technology, Eindhoven, The Netherlands; 5 Department of Values, Technology and Innovation, Delft University of Technology, Delft, The Netherlands

**Keywords:** AI, Algorithm, Hiring, Autonomy, Self-representation, Dignity

## Abstract

In this paper, we examine the epistemological and ontological assumptions algorithmic hiring assessments make about job seekers’ attributes (e.g., competencies, skills, abilities) and the ethical implications of these assumptions. Given that both traditional psychometric hiring assessments and algorithmic assessments share a common set of underlying assumptions from the psychometric paradigm, we turn to literature that has examined the merits and limitations of these assumptions, gathering insights across multiple disciplines and several decades. Our exploration leads us to conclude that algorithmic hiring assessments are incompatible with attributes whose meanings are context-dependent and socially constructed. Such attributes call instead for assessment paradigms that offer space for negotiation of meanings between the job seeker and the employer. We argue that in addition to questioning the validity of algorithmic hiring assessments, this raises an often overlooked ethical impact on job seekers’ autonomy over self-representation: their ability to directly represent their identity, lived experiences, and aspirations. Infringement on this autonomy constitutes an infringement on job seekers’ dignity. We suggest beginning to address these issues through epistemological and ethical reflection regarding the choice of assessment paradigm, the means to implement it, and the ethical impacts of these choices. This entails a transdisciplinary effort that would involve job seekers, hiring managers, recruiters, and other professionals and researchers. Combined with a socio-technical design perspective, this may help generate new ideas regarding appropriate roles for human-to-human and human–technology interactions in the hiring process.

## Introduction

The use of artificial intelligence (AI) algorithms for assessment of job seekers’ attributes and job fit is becoming more common across different industries (Bogen and Rieke 2018; Crawford et al. 2019). These assessments come in a variety of forms, for example automated online interviews and games, that apply machine learning to map candidates’ responses and behaviors to a wide range of attributes, such as willingness to learn, relationship building, generosity, and decision making (Mondragon et al. 2021; Pymetrics, n.d.). One big vendor of such assessments, HireVue, refers to measured attributes as “competencies,” “cognitive ability,” “skills,” “personality traits,” and “emotional intelligence” (Mondragon et al. 2021: 17). Other vendors, like pymetrics and Harver, describe the job seeker attributes they assess as “soft skills” and “cognitive and emotional attributes” (Harver, n.d.; Pymetrics, n.d.). Given the variety of terms vendors use to describe what kind of attributes are measured, in this paper we will refer to these more generically as *job seekers’ attributes*. From these attributes, some algorithms then make a further inference about job fit. HireVue, for example, defines job fit as “the optimal combination of personality traits, cognitive ability, and competency areas for a target set of job roles” (Mondragon et al. 2021: 5).

Vendors of algorithmic assessments promise a range of benefits that are highly attractive to employers (Li et al. 2021). One common benefit claimed by vendors is that their assessments can save many hours of tedious and costly work, allowing recruiters and hiring managers to focus their efforts on interviewing the best candidates for the job (Harver, n.d.; HireVue, n.d.; Modern Hire, n.d.). Furthermore, vendors often claim that their algorithms make assessments that substantially reduce or even fully eliminate individual prejudice and systematic bias, contributing to fairness, diversity, and inclusion in the hiring process (Drage and Mackereth 2022; Raghavan et al. 2020).

However, the use of algorithmic hiring assessments and the bold claims made by their vendors have come under increasing ethical, legal, and scientific scrutiny. The ethical and legal aspects that seem to have received most attention in academic literature thus far are algorithmic bias and discrimination (Ajunwa 2021; Bogen and Rieke 2018; Hunkenschroer and Luetge 2022; Raghavan et al. 2020; Sánchez-Monedero et al. 2020). While these issues are very important, additional and fundamental concerns have been raised about the validity of these assessments and the claims being made. Some industrial and organizational (I–O) psychologists have expressed concerns about the scarcity of available evidence supporting the validity, reliability, and fairness of these tools (Gonzalez et al. 2019; Tippins et al. 2021). In fact, a recent study auditing two personality-assessing algorithms used in hiring concluded that both tools failed to exhibit sufficient reliability and therefore cannot be considered as valid assessments (Rhea et al. 2022). Furthermore, some algorithmic assessments measure features for which there is no established and scrutinized theory relating them to job seeker attributes or job performance (e.g., features like tone of voice and facial expressions) (Ajunwa 2021; Hinkle 2021; Stark and Hutson 2021; Tippins et al. 2021). Sloane et al. (2022) have argued that it is therefore critical to step back and examine the assumptions underlying the use of AI algorithms in the hiring process.

In this paper, we contribute to this effort by conceptually analyzing the epistemological and ontological assumptions algorithmic hiring assessments make about job seekers’ attributes and the ethical implications of these assumptions. Hiring assessments can be viewed as tools for producing knowledge about the job seeker, with the goal of informing the hiring decision-making process. As knowledge production tools, they embody certain epistemological and ontological assumptions that together constitute a knowledge production paradigm. But what is that paradigm and what are the assumptions it makes? Which meanings of job seekers’ attributes are discoverable through this paradigm and which are missed? Which alternative paradigms can be considered? And, what are the ethical implications of these paradigm choices? We begin our conceptual analysis, by observing that both traditional hiring assessments and AI-based hiring assessments share a common set of underlying epistemological and ontological assumptions from the psychometric paradigm. Based on desk research, we then turn to literature that has examined the merits and limitations of these assumptions, gathering insights across multiple disciplines and several decades. By doing so, we invite the readers to join us on a journey of connecting the dots between insights from the past and questions posed about technologies of today.

Our exploration leads us to conclude that algorithmic hiring assessments are incompatible with attributes whose meanings are context-dependent and socially constructed. Such attributes call instead for assessment paradigms that offer space for negotiation of meanings between the job seeker and the employer. We argue that in addition to questioning the validity of algorithmic hiring assessments, this raises an often overlooked ethical impact on job seekers’ autonomy over self-representation: their ability to directly represent their identity, lived experiences, and aspirations. In our view, this key aspect of human dignity deserves as much attention within AI ethics as the more prominent topics of non-discrimination (fairness), explainability, and privacy.

We conclude with a suggestion to begin addressing these issues through epistemological and ethical reflection regarding the choice of assessment paradigm, the means to implement it, and the ethical impacts of these choices. This entails a transdisciplinary effort that would involve job seekers, hiring managers, recruiters, assessment experts, design researchers, ethicists of technology, and other professionals who collectively with stakeholders study the work context, identify and navigate epistemological and ethical tensions, and co-design the assessment process. Combined with a socio-technical design perspective, this may help generate new ideas regarding appropriate roles for human-to-human and human–technology interactions in the hiring process.

## What are the assumptions?

Algorithmic hiring assessments produce knowledge about the job seeker by measuring the job seeker’s responses to certain stimuli (e.g., measuring speech features in response to questions in an automated interview, or measuring game behavior in a gamified assessment). This may sound as a very abstract description that ignores the technical details of what is measured, how it is measured, and how the measurements are processed. But this description actually represents an epistemological choice of how knowledge is produced: measurement of responses to stimuli. On this basic level of analysis, algorithmic assessments share a common epistemology with traditional psychometric hiring assessments. It is certainly true that the types of stimuli, measurements, and the technical methods of mapping those measurements to knowledge in the form of assessment scores can be very different when comparing traditional assessments and AI-based assessments (Liem et al. 2018). To be clear, here we want to focus on the underlying epistemological and ontological assumptions of the psychometric paradigm, which are shared by both traditional and algorithmic hiring assessments (Rhea et al. 2022). This shared set of underlying assumptions implies that their merits and limitations—which have been explored in academic literature over multiple decades—also apply to algorithmic hiring assessments. Therefore, we now turn to some of this literature to examine what are the assumptions these assessments make about job seekers’ attributes, which meanings of job seekers’ attributes are discoverable through such assessments, and which meanings are missed.

A key assumption under the psychometric assessment theory is that “the person’s knowledge, attitude, skill, or other measured attribute is a steady state; that is, we assume that any differences among scores earned by an individual on different occasions of measurement are due to one or more sources of error, and not to systematic changes in the individual due to maturation or learning” (Shavelson and Webb 1991: 1). There are a number of aspects worthwhile to highlight more explicitly here. Based on elaborate examination of this matter by Delandshere and Petrosky (1998) and Govaerts and Van der Vleuten (2013), we point out that psychometric assessment theory assumes that:

1. The person’s measured attribute is stable across time and contexts.
2. There exists a true attribute value to be measured, i.e., the true score.
3. Variability in measurements of a person’s attribute across time and contexts is due to measurement error (noise).
4. The assessed attribute can be meaningfully represented by a numerical scale.

Some of the rationale behind the stability across time and contexts assumption is that an individual’s scores should be similar if they were assessed on multiple occasions, and that scores should not be affected by factors like day or time of assessment, the equipment used, and context, unless these factors are job relevant (Tippins et al. 2021). In this sense, the true performance value (the true score) is an idealization that refers to the average score a person would receive if tested under all possible acceptable conditions (Shavelson and Webb 1991).

But which meanings of job seekers’ attributes are discoverable through such knowledge production paradigm and which are missed? The assumption that there exists a single, true attribute value to be measured implies that there is a universally true answer to the question of how much of that attribute (e.g., competency, skill, ability) does the job seeker possess, independent of context and time. Under this assumption, it is plausible that the assessed attribute can be meaningfully represented by a numerical scale. As Delandshere and Petrosky (1998: 23) point out, “numerical ratings are useful in representing occurrences of simple and discrete behaviors that manifest themselves consistently across individuals, contexts, and time and where the correspondence between the assignment of ratings and the observed behaviors is more obvious.” It is fairly straightforward to see how, for example, the skill of lifting a specific weight or running a certain distance in a given time, can be numerically rated based on how close the job seeker’s performance is to the target value. In these cases, there is a clear correspondence between numerical ratings that measure weight, distance, or time, on the one hand, and the observed performances and their meaning, on the other. And within some restricted period of time, variability across multiple performances and contexts can be seen as variations around some true level of ability.

Let us now consider a different type of attributes, for example teamwork and creativity. In many contexts, the meanings of these attributes are not reducible to simple, discrete behaviors that are consistent across individuals, contexts, and time. On the contrary, we might be looking for aspects of job performance that are unique to that individual and whose meaning is a product of an individual’s interaction with others (e.g., colleagues and clients) in a specific work setting and socio-cultural context (Govaerts and Van der Vleuten 2013). The way a person expresses teamwork or creativity can vary in different contexts. There is then no single true value for creativity and teamwork that is stable across contexts and time, but rather a plurality of expressions whose meanings are constructed by an individual and the people they interact with in the surrounding context. Note that for such attributes, variability across contexts and time should not be dismissed as an “error” or “noise”.^1^ Instead, it is integral to appreciating the individual and the specific ways they can contribute to a job (Delandshere and Petrosky 1998; Govaerts and Van der Vleuten 2013).

Attributes whose meanings are context-dependent and socially constructed are incompatible with a knowledge production paradigm that measures responses to stimuli in search of a single true answer. The assumption that there is one true answer eliminates the possibility of multiple true answers. Production of knowledge through measurement of responses to stimuli does not leave room for negotiation of meanings among people. Which alternative paradigms of knowledge production could be helpful here? As argued by a number of authors, social constructionist and interpretivist paradigms are well suited to capture such plurality, context-dependent nuance, and offer space for negotiation of meanings (Govaerts and Van der Vleuten 2013; Pratt and Bonaccio 2016; Tafreshi et al. 2016). This often entails *qualitative research* aimed at understanding *what*, *how*, and *why* individuals are doing or have done in a particular context. Importantly, in this process the employer would make an effort to view the world from the perspective of the job seeker (Bryman 1984). Interpretive assessment “focuses on participants’ own perspectives in conceptualizing and reconstructing their experiences and world view” (Gipps 1999: 371). In turn, this provides the job seeker the ability to engage in direct representation and storytelling about their lived experiences and aspirations.

Despite the incompatibility of the psychometric paradigm with attributes whose meanings are context-dependent and socially constructed, both traditional and AI-based hiring assessments apply this paradigm to measure such attributes. Doing so, restricts and reduces the meaning of the assessed attribute to a non-negotiable numerical scale. In effect, the meaning of the attribute becomes defined by the assessment (Lantolf and Frawley 1988; Vollmer 1981). Delandshere and Petrosky (1998: 17) point out that this circular thinking has been a common characteristic of psychometric assessments: “[c]onstructs were not defined theoretically, but instead on the basis of the tests that served to measure them and on the statistical methods used to analyze the scores they yielded.” This has root in the widely accepted definition of measurement in psychology, viewing measurement as “the assignment of numerals to objects or events according to rules” (Stevens 1946: 677). As Tafreshi et al. (2016: 238) explain, such a flexible definition “leaves no room for questioning the adequacy of those numbers for capturing the nature of psychological attributes.” Michell (1997, 2000, 2003) has argued that it has become common practice in psychology to quantify psychological attributes without presenting evidence that these attributes have quantitative properties, but rather merely presuming that they do (Tafreshi et al. 2016).

Given the incompatibility of the psychometric paradigm with attributes whose meanings are context-dependent and socially constructed, we question the validity of algorithmic hiring assessments as tools for producing knowledge about such job seeker attributes. An unreflective application of these assessments can result in distortion and loss of crucial information about job seekers. This not only puts into question the validity of algorithmic hiring assessments, but also raises an often overlooked ethical impact on job seekers’ autonomy and dignity, which we explore below.

## Ethical impact: autonomy over self-representation

Algorithmic hiring assessments impose a reductionist, non-negotiable conception of job seekers’ attributes. This knowledge production paradigm eliminates the possibility for the job seeker and the employer to negotiate an aligned knowledge representation about *what*, *how*, and *why* the job seeker is doing or has done in a particular context, and how that informs their suitability for the job. The job seeker is denied of what Bernard Williams (1973: 236) called an “effort at identification: that [a person] should not be regarded as the surface to which a certain label can be applied, but one should try to see the world (including the label) from [the person’s] point of view.” We suggest that what is at stake here is a key aspect of the job seeker’s dignity and autonomy: their ability to act as a direct representative and storyteller of their identity, lived experiences, and aspirations, while acting as active constructors of the representations through which others view them. Building upon related concepts, we refer to this aspect of human dignity as *autonomy over self-representation*.

Halbertal (2015) has discussed the notion of control over self-representation as a key dimension of human dignity, referring to a person’s autonomy to represent themselves to the world the way they wish to. This kind of autonomy is often at stake in the context of privacy, specifically an individual’s control over what private information about them is shared with others. The common concern invoked in this context is exposure of private information that the person did not wish to share, undermining their standing as a social agent (Velleman 2005). The complementary aspect of self-representation we are focusing on here involves the things an individual considers essential to share or display to represent their identity, lived experiences, aspirations, etc. (Risam 2018). These are aspects of the self that cannot be fully known or understood by outside observation or measurement because these would fail to engage with the individual’s own perspective on their life (Manders-Huits and Van den Hoven 2008). Therefore, we see autonomy over self-representation as not only the ability to choose what to share or display to the world, but also the ability of a person to engage with the world to construct and negotiate the representations through which others view them.

**Fig. 1:**
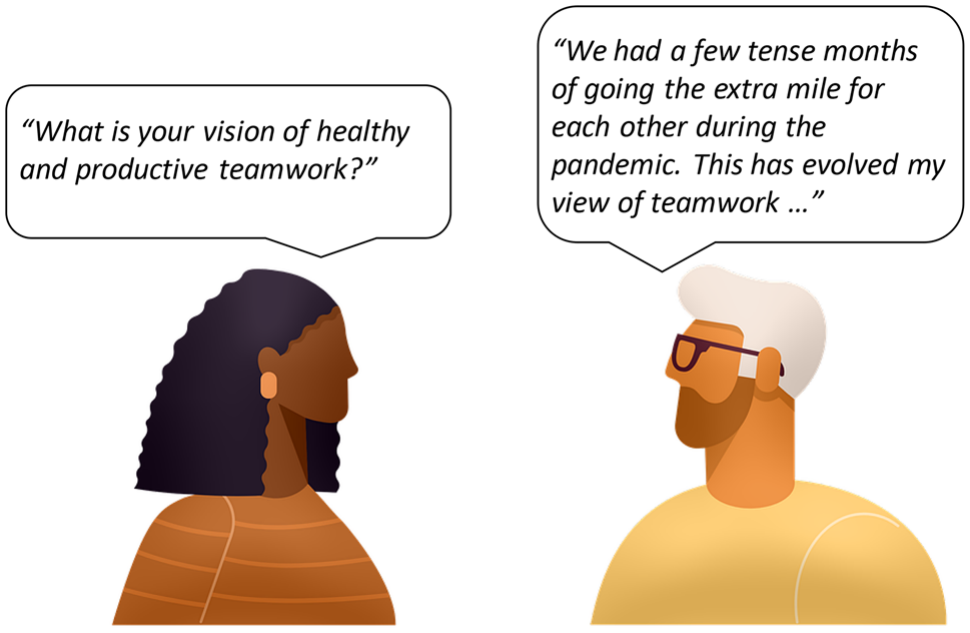
Traditional face-to-face interview. The job seeker directly represents themselves, their experiences, skills, and aspirations to the employer. Meanings are actively negotiated through conversation. (Artworks adapted from Pixabay)

**Fig. 2:**
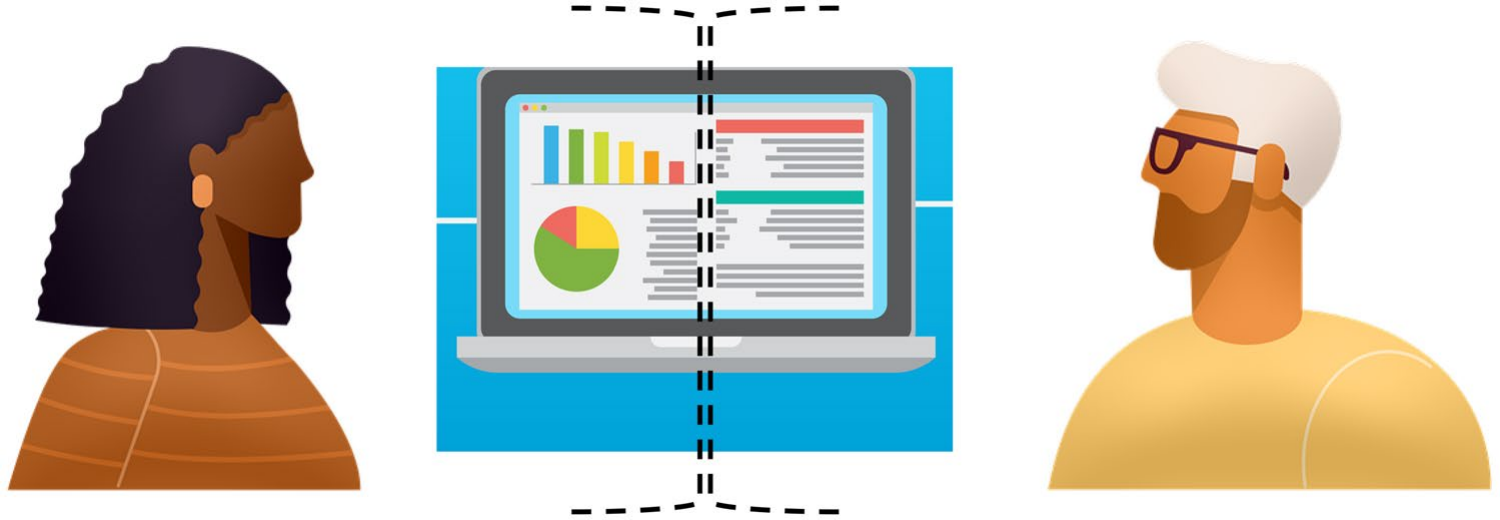
AI-based interview. The algorithmic assessment constructs a numerical representation of the job seeker in the form of scores, based on the measured features of their recording. (Artworks adapted from Pixabay)

Ultimately, hiring decisions are based on a representation of the job seeker and their attributes. These representations may, for example, involve some information about the job seeker’s past experiences, knowledge, skills, and aspirations. Let us examine how such representations are constructed in two contrasting scenarios: a traditional job interview in which the job seeker (Paul) and the employer (Michelle) meet face to face (Fig. 1) and an AI-based interview in which Paul records answers to questions posed by an algorithmic assessment (Fig. 2). The presented scenarios are quite schematic, but we believe they can help appreciate the impacts of different paradigms of assessment on autonomy over self-representation on a more intuitive level.

### Scenario 1: face-to-face interview

During a face-to-face interview, Michelle asks Paul what is his vision on healthy and productive teamwork. Paul replies by sharing a recent experience in which several of his team members became ill during the COVID-19 pandemic, and how he and his colleagues navigated a few tense months of work pressure by filling in for each other while collectively struggling to keep up with the workload. While Paul has no guarantee that Michelle interprets this experience the same way he does, the face-to-face interaction provides Paul with room for feedback and negotiation. He can semantically negotiate with Michelle and strive to align the mutually perceived meaning of what he wishes to communicate: the significance of that experience to him, and how it has influenced his approach to working in teams. Note that although perfect alignment in meaning may be difficult or even impossible to achieve, Paul is acting as a direct representative of himself and is actively negotiating with Michelle the meaning of his experiences.

### Scenario 2: AI-based interview

In the AI-based interview, Paul records answers to questions posed to him by an algorithmic assessment. After the recording is submitted, the algorithm scans the video for Paul’s facial expressions, choice of words, and voice tone. Based on these measured features, the algorithm computes numerical scores for teamwork, willingness to learn, and conscientiousness, which are later presented to Michelle along with scores of other candidates. During the interview, Paul has no indication how the algorithm interprets his answers. Although he recounts the pandemic-related experience and its significance to his approach to teamwork, the algorithm is actually not capable of interpreting that information in a way that can capture its qualitative richness, context, and meaning. Thus, an experience Paul considers essential to his self-representation and suitability for the job is missing from the representation produced by the algorithm. In fact, Michelle may never hear Paul’s story, unless she explicitly decides to view his recording. But for that to happen, the scores the algorithm assigned to Paul need to be high enough so that he is among the top-ranked candidates.

Although schematic, these contrasting scenarios provide some intuition on how the choice of assessment paradigm affects job seekers’ autonomy over self-representation. While in both cases Paul has the ability to share his story, the AI-based interview does not provide Paul with any possibility to negotiate the numerical representation the algorithm constructs and the meanings it communicates to Michelle. This is in stark contrast to the face-to-face interview where Paul and Michelle actively negotiate meanings between each other. The teamwork score presented to Michelle reduces Paul’s lived experiences to a single number void of qualitatively nuanced storytelling he tried to communicate. Furthermore, the scores may obscure the fact that the job seeker is a dynamic person who engages in self-improvement and evolves over time in ways that are not deterministic (Govaerts and Van der Vleuten 2013; Manders-Huits and Van den Hoven 2008). Each of these factors poses a real risk that the job seeker will be judged based on what the *algorithm says* they are capable of, rather than what the *job seeker says* they are capable of or plan to do, an example of what has been informatively labeled as “data determinism” (Ramirez 2013).

## Implications for research and practice

In this paper, we sought to contribute to the effort of examining the knowledge production assumptions of algorithmic hiring assessments and their ethical implications. We believe that a key takeaway from this initial exploration is that job seekers’ attributes whose meanings are context-dependent and socially constructed are incompatible with an assessment paradigm that searches for a single true answer based on measurement of responses to stimuli. Such attributes call for assessment paradigms that offer space for negotiation of meanings between the job seeker and the employer. The absence of ability to negotiate meanings brings us to recognize that what is at stake is not only the validity of AI-based assessments but also their ethical impact on job seekers’ autonomy over self-representation, a key dimension of human dignity. The ability of the job seeker to act as a direct representative and storyteller of their identity, lived experiences, and aspirations is critical for arguing why they believe they are a good candidate for a job. And this autonomy is especially important for making their case about attributes whose meanings are context-dependent and socially constructed. Respect for job seekers’ autonomy over self-representation calls upon employers to make an effort to see the world from the job seekers’ perspective.

While we focused on assumptions algorithmic hiring assessments make about job seekers’ attributes, we did not address epistemological assumptions that underpin the mapping of measurements to scores. However, it is important to note that these mappings also have profound impact on autonomy over self-representation through the inherent comparison of the job seeker to the population sample these algorithms were trained on. This imposes a conception under which the job seeker is de-individualized (Vedder 1999): What matters is not the unique aspects of their attributes, but how the quantified aspects of their attributes compare to the “competent candidates” in the population sample the algorithm was trained on.

Looking for a moment beyond the hiring domain, we believe that autonomy over self-representation is an overlooked ethical issue that deserves as much attention within AI ethics as the more prominent topics of non-discrimination (fairness), explainability, and privacy. AI algorithms are being applied to produce knowledge about people in other high-stakes domains of life, such as healthcare, policing, finance, and welfare. These algorithms construct representations of individuals through which others view them and make decisions that affect their lives. As explored here, this can impose a reductionist, non-negotiable conception of the person, taking away their ability to construct the representations through which others view them. Furthermore, the quantitative and automated nature of algorithms can falsely create an impression of objectivity regarding the representations they produce. Therefore, we believe there is an urgent need to take a step back to reflect on what is an appropriate knowledge production paradigm for a given context, the means to implement it, and what are the ethical implications of these choices, including impact on individuals’ autonomy over self-representation. Reflection on knowledge production assumptions is also relevant for other constructs AI algorithms attempt to measure, for example fairness. Such reflection would complement the exploration of theoretical understandings and measurement assumptions about fairness discussed by Jacobs and Wallach (2021) by examining whether measurement itself is epistemologically compatible with contextual meanings of fairness, or whether a different paradigm for producing knowledge is warranted. In the remainder of this section, we share some initial thoughts on how these serious issues could be addressed in the design of hiring assessments going forward.

We suggest to engage in epistemological and ethical reflection regarding the choice of knowledge production paradigm before a decision is made on pursuing an algorithmic approach to assessment. This reflection would seek to contextually investigate the kind of questions we asked at the beginning of this paper:

1. Which meanings of job seekers’ attributes are discoverable through a given paradigm and which are missed?
2. Which alternative paradigms can be considered?
3. What are the ethical implications of these paradigm choices?
4. Which means of assessment are compatible with the preferred paradigm?

One of the critical first steps in this reflection would be to align the understanding of contextual meanings of various attributes among job seekers, hiring managers, recruiters, and designers and developers of assessments. This requires a qualitatively rich empirical investigation that together with these stakeholders reveals the often implicit assumptions of what attributes a given job entails and which qualities characterize a good job candidate. Such investigation would not suffice itself with establishing that “teamwork” is an important attribute, but would engage stakeholders to further specify what teamwork entails in the context of that job through concrete examples, storytelling, and deliberation among stakeholders. In this process of making the implicit explicit, the investigation may reveal areas where stakeholders wrongly assumed to have consensus among each other when, in fact, significant misalignments in meanings are present (Van den Broek et al. 2019). It is crucial to emphasize the importance of involving job seekers in this process. As early studies on autonomy over self-representation in hiring illustrate, it is this kind of direct engagement with job seekers that brings to the surface specific needs that can inform design choices (Ter Haar Romenij 2020; Van der Ploeg 2021).

Having established the contextual meanings of job seekers’ attributes, it is possible to begin exploring which assessment paradigms are compatible with capturing these meanings and how to implement them. At this step, it is essential to keep an open mind regarding what the roles of humans and algorithms might be, avoiding technical solutionism (Morozov 2013; Selbst et al. 2019) and assuming upfront that algorithms must be part of the assessment process. If the empirical investigation indicates that the meanings of a specific attribute align with the assumptions of the psychometric paradigm (i.e., existence of a single true value, variations over context and time being measurement error, etc.), then the use of psychometric measurement can be considered as possible means of assessment for that attribute. On the other hand, an attribute whose meanings are context-dependent and socially constructed would call for assessment paradigms that offer space for negotiation of meanings. This entails interaction between the job seeker and the employer. Such interaction can take the form of a face-to-face conversation, but it does not have to be the only meaningful way to achieve it. Furthermore, human-to-human interaction alone is not itself a guarantee that the assessment is not reductionist, as humans are certainly prone to making unfounded assumptions about each other in ways that can harm autonomy over self-representation. This human-to-human interaction needs to be embedded within a larger system with organizational process, policy, and training for hiring managers and recruiters, which collectively act in support of job seekers’ autonomy over self-representation. In fact, digital technology (not necessarily AI, but possibly as well) may be able to support and facilitate such interactions in innovative and meaningful ways. But note that this entails a very different role for digital technology compared to the dominant narratives marketed by vendors of AI-based hiring assessments. Instead of replacing human-to-human interaction with automated assessments, the focus of the technology would be supporting human-to-human interaction. This highlights the need for a socio-technical design perspective that jointly considers *human-to-human* and *human–technology* interactions.

Carrying out the outlined reflection process and empirical investigation is not an exercise that a single profession can engage in alone. It requires integrating ways of knowing brought by different stakeholders, professional fields, and academic disciplines—a transdisciplinary team effort. Such an effort would involve job seekers, hiring managers, recruiters, assessment experts, design researchers, ethicists of technology, and other professionals who collectively with stakeholders study the work context, identify and navigate epistemological and ethical tensions, and co-design the assessment process. Van der Bijl-Brouwer (2022: 9) identifies “epistemic intelligence, worldview awareness, power literacy, and reflexive and dialogic skills” as important competencies for engaging in transdisciplinary work.

One may point out that even with sincere intentions and efforts of all stakeholders to design for autonomy over self-representation, there is a fundamental tension between the needs of job seekers and the resources of employers. Even with support of digital technologies, it is likely that designing for autonomy over self-representation would require employers to invest more human effort, time, and budget into the hiring process. For jobs where the volume of applicants is high, this tension is likely to be especially pronounced. While limitations to job seekers’ autonomy are likely to occur in this balancing act, any derogation of job seekers’ autonomy over self-representation requires a strong justification and scrutiny. Based on the exploration presented in this paper, we argue that both scientifically and morally it is unacceptable to use hiring assessments that impose a reductionist, non-negotiable view on job seekers’ attributes whose meanings are context-dependent and socially constructed. Resolving these larger-scale tensions is a serious challenge that takes us beyond the scope of this paper. These dilemmas tie into systemic questions concerning the labor market, the economy, and ultimately political choices a society makes. However, an explicit recognition of these tensions, as well as the fact that it is often not possible to simply “tech” our way out of them, is a first step toward an honest conversation that could give rise to practical improvements.

## Conclusion

The knowledge production paradigm behind algorithmic hiring assessments assumes that job seekers’ attributes are stable over time and contexts, and that they can be assessed by measuring job seekers’ responses to various types of stimuli. Our exploration of past insights on the merits and limitations of these psychometric assumptions led us to conclude that they are incompatible with job seekers’ attributes whose meanings are context-dependent and socially constructed. Such attributes call instead for assessment paradigms that offer space for negotiation of meanings between the job seeker and the employer. We have argued that in addition to questioning the validity of algorithmic hiring assessments, this raises an often overlooked ethical impact on job seekers’ autonomy over self-representation: their ability to directly represent their identity, lived experiences, and aspirations. Algorithmic hiring assessments undermine job seekers’ ability to construct and negotiate the representations through which the employer views them. This infringement on autonomy over self-representation constitutes an infringement on job seekers’ human dignity.

We suggest beginning to address these issues through epistemological and ethical reflection regarding the choice of assessment paradigm, the means to implement it, and the ethical impacts of these choices. This entails a transdisciplinary effort that would involve job seekers, hiring managers, recruiters, and other professionals and researchers. In this process, it is essential to keep an open mind about the possible roles of humans and technology and not assume upfront that algorithms must be part of the assessment process. Combined with a socio-technical design perspective, this may help generate new ideas regarding appropriate roles for human-to-human and human–technology interactions in the hiring process, which may differ substantially from the dominant narratives of today.

## Data Availability

Data sharing is not applicable to this article as no datasets were generated or analyzed during the current study.

## Footnotes

1 For a spirited defense of the attractiveness of AI systems for reducing undesirable variability in human judgement, see Daniel Kahneman et al.’s recent book: *Noise: A Flaw in Human Judgment* (2021).
